# Provider bias and family planning in Upper Egypt: a simulated client approach

**DOI:** 10.1186/s42506-023-00144-6

**Published:** 2023-10-01

**Authors:** Mirette M. Aziz, Amira F. El-Gazzar

**Affiliations:** 1 Department of Public Health and Community Medicine, Assiut University, Assiut, Egypt; 2 Department of Public Health and Community Medicine, Badr University in Cairo, Badr City, Egypt

**Keywords:** Family planning, Provider, Bias, Egypt

## Abstract

**Background:**

Provider bias is a main barrier that extensively violates the right of free family planning method choice. Egypt is one of the countries that shows skewness in its method mix. Provider bias and insufficiency of alternative methods are identified as potential factors underlying this phenomenon which contributes to high unmet needs and discontinuation rates. Provider bias may be influenced by cultural beliefs and societal trends and is usually overlooked as a possible cause of this skewed method mix. This study aims to explore the presence of provider bias in rural Upper Egypt and its potential causes, a community with conservative cultural beliefs and least contraceptive prevalence rates.

**Methods:**

This is a qualitative study using the “simulated client’s approach.” The study was conducted in 16 villages in Assiut and Sohag governorates in Egypt. The simulated clients visited 30 clinics, 15 in each governorate, including primary healthcare units and private clinics. Three scenarios were used to explore the physicians-imposed restrictions for contraceptive use with different clients’ eligibility criteria. Data was analyzed using the grounded theory methodology.

**Results:**

Recommending a contraceptive method for the mystery clients was not based on informed choice. Most providers had method or client bias. Copper IUD was the most favorable contraceptive method recommended by providers, with negative attitude towards using hormonal contraception. Nulliparous and young clients were discouraged to use contraception before proving fertility or offered temporary methods as emergency contraception or condoms. Providers have shown misconceptions related to infertility-associated complications of contraceptive use, especially for the young and nulliparous women.

**Conclusion:**

In this study, providers had a clear bias towards recommending IUD rather than all other contraceptive methods, which was hindered in some cases by the lack of insertion skills. Interventions to reduce provider bias should go beyond technical training. Moreover, training on reproductive rights should be a main component of routine training. Providers should regularly receive research results and be oriented toward recent medical eligibility criteria of contraceptive methods use. Moreover, the sociocultural beliefs of providers that may affect their practice should be explored and addressed.

## Introduction

Strengthening family planning services is crucial for improving health, human rights, and economic development and slowing population growth [1]. Family planning programs are based on the principle of informed choice as well as providing a broad choice of contraceptive methods to meet the diverse needs of clients [2]. Family planning clients have the right to be fully informed on the benefits, potential adverse effects, and available alternatives by trained personnel. The health system is responsible for providing proper and noncoercive counseling, accurate information, and competent providers [3].

Involving clients in decisions about their health care specially their reproductive health increases clients’ confidence, commitment, and satisfaction [4]. On the other hand, inadequately informed clients face higher post-use health problems, discontinuation, unmet need for contraceptives, unintended pregnancies, and induced abortions [5].

However, a number of barriers limit an individual’s access and actual choice, including both supply and demand factors, leading to a large proportion of women in reproductive age with unmet needs for modern contraception. Most of the women with an unmet need for contraception live in the poorest countries, with a high documented rate in Egypt reaching 13% of women in reproductive age [6].

Provider bias is a main barrier that extensively violates the right of free family planning method choice. It is defined as “attitudes and subsequent behaviors by providers that unnecessarily restrict client access and choice, often related to either client and/or contraceptive method characteristics” [7]. Provider bias and related practices are usually based on the demographic and obstetric profile of the clients such as age, parity, and marital status. Some providers are influenced by their culture and social norms in addition to their moral and personal values when they counsel their clients [8]. Various studies have shown that large numbers of providers impose barriers and restrictions beyond those conveyed in normative guidelines or needed for any medical reasons [7].

Egypt is one of the countries that shows skewness in its method mix, as intrauterine device (IUD) is used by more than half of family planning users, while the other half use all the other methods [9]. This pattern indicates high possibility of existence of provider bias that could be related to cultural beliefs and societal trends or point to insufficiency of alternative methods [10]. Provider bias is usually overlooked as a possible cause of this skewed method mix, which in turn contributes to higher unmet needs and discontinuation rates. Egypt is recently facing an increase in discontinuation rates mainly due to method-/service-related reasons [11]. This study aims to explore the presence of provider bias in rural Upper Egypt, a community with conservative cultural beliefs and least contraceptive prevalence rates. It aims also to identify the potential causes of bias, which is essential for improving family planning programs and promoting contraceptive use.

## Methods

### Type of the study

This is a qualitative study using the “simulated client’s approach.” This approach was selected to measure the real interaction between clients and physicians and the spontaneous response to clients inquires [7].

### Study sites

The study was conducted in 16 villages in Assiut and Sohag governorates. Assiut and Sohag were chosen because they have the least recorded contraceptive prevalence rates in Egypt [9]. The simulated clients visited 30 clinics, 15 in each governorate, including primary healthcare units and private clinics.

### The study tools

Three scenarios were developed for the simulated clients, to explore the physicians-imposed restrictions on contraceptive use with clients who have varying eligibility criteria. The first scenario was about a nulliparous client younger than 20 years old. The client in the second scenario was a 30 years old woman who had a history of pelvic inflammatory disease (PID). The third one was about a client who expressed her worries regarding the IUD-related menstrual disturbances, as heard from her relatives and friends. Clients in the second and third scenarios claimed to have one or two children. The different scenarios were designed to capture potential variations in how providers would respond to women with different needs and the provided counseling in different situations.

For all scenarios, the simulated clients were asked to report on several aspects, including the provider-client interaction, the general and obstetric history obtained, the way of responding to the client’s inquiries, the provider’s recommended method, and information provided by the provider about the recommended method (including advantages, disadvantages, and side effects). They were also asked to report on the provider’s comments regarding age of the client, the existence of a history of PID, whether the provider mentioned other contraceptive methods, and whether the final choice was made by the client.

### Data collection

Four data collectors were trained on the scenarios of simulated clients. In each visit, one simulated client pretended to ask for a contraceptive method, and the other one simulated an accompanying relative. They dressed, spoke, and behaved as if they were usual family planning clients at a rural health facility. The simulated clients were trained to only ask the provider for a contraceptive method and receive consultation without being subjected to any examination or clinical procedures, by showing hesitancy to use the offered method and the desire to refer to their husbands for having a joined decision-making.

The simulated client recorded their observations after each visit, wrote a transcript immediately after the visit, and had a debriefing session with the researchers at the end of each day of data collection.

### Data analysis

The visits of mystery clients were transcribed, and a thematic analysis was conducted to obtain in-depth understanding of provider bias. The grounded theory methodology was used for data analysis. The transcripts were read repeatedly, and the raw data was thematically coded. Codes and labels were attached to text parts related to a specific theme, resulting in a set of descriptive themes and subthemes per transcript. Next, all codes were grouped into themes and subthemes. The themes were confirmed, modified, or discarded during ongoing analysis through re-examination of earlier data and subsequent data collection.

## Results

### Background characteristics

A total of 30 physicians were visited by the simulated clients in the study including 19 males and 11 females. The age of the physicians ranged between 27 and 58 years old. Thirteen visits were performed in primary healthcare units including seven visits in rural Assiut and six visits in rural Sohag. Seventeen visits were performed in private clinics in both governorates.

### Method-related bias

#### Recommending a contraceptive method was not based on informed choice

**Fig. 1:**
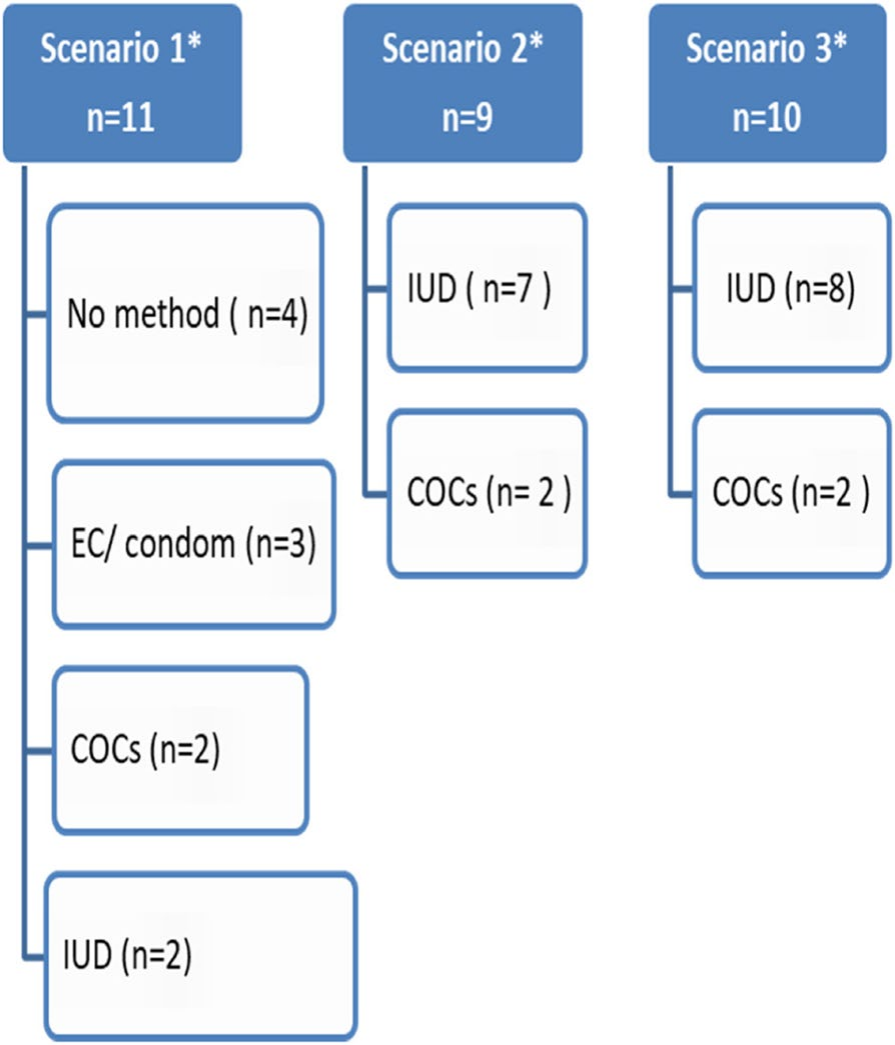
Flow chart showing the methods recommended in the different scenarios. *Scenario 1, a nulliparous client younger than 20 years old; scenario 2, a 30-year-old woman who had a history of PID; scenario 3, a client who expressed her worries regarding the IUD-related menstrual disturbances. IUD, intrauterine device; EC, emergency contraception; COCs, combined oral contraception. *According to the WHO medical eligibility criteria, the 3 scenarios lie in category 1 “which is using any of the contraceptive methods with no restrictions in these circumstances (the clients should have been offered all the family planning methods in the 3 scenarios) [12]

During almost all visits, it was observed that providers recommended either copper IUD or contraceptive pills (Fig. 1). And none of the clients was offered hormonal implants, injectables, or any barrier methods. The decision-making process was mostly controlled by the providers, and the clients were not given an informed choice. Moreover, providers appeared to offer clients limited choices of family planning methods and usually directing clients towards a particular method (usually copper IUD) and disparaging other methods. Providers were reluctant in offering proper counseling, including information on the advantages and disadvantage of each method, how it works, side effects, duration of use, and effect on sexual relation. Almost all provided information was in response to patients’ (i.e., simulated client) inquiries and not always adequately addressed.

#### Copper IUD was the most favorable contraceptive method recommended by providers

It was observed that IUD was the most frequently recommended method for simulated clients (17 clients out of 30; by 8 physicians in PHC units and 9 in private clinics), except for the nulliparous ones (Fig. 1). Providers primarily focused on describing its advantages and did not mention any expected side effects. Moreover, they have avoided mentioning other suitable methods, to prevent clients from being distracted from their recommended use, or in some cases, they mentioned the side effects of other methods and exaggerate its disadvantages to convince the clients to use the IUD.

Being a nonhormonal method was the main driver of recommending IUD by most physicians as they consistently mentioned this information to the clients. Another reason for recommending IUD use to the simulated clients was lack of side effects; such as no impact on fertility, weight gain, or hypertension. Additionally, some physicians highlighted the advantages of IUDs including their long-term effectiveness and the fact that they do not require any intervention on the part of the client.

The following are some quotes of physicians’ counseling.

*Female physician, 58 years old, PHC unit:*

If you want me to recommend you the best method,,, It is definitely IUD,, a safe method that you do not need to remember or take any precautions after its insertion,, you would not then say; I forgot to take the pill, I got tired after the injection, I got pregnant while using pills.

*Female physician, 47 years old, PHC unit (scenario 1):*

IUD does not cause any problems, and it is a local method, you can remove anytime and get pregnant easily, it does not cause ovarian cysts, nor hormonal disturbances, it is the safest method, you just get it inserted and forget about.

*Female physicians, 37 years old, private clinic:*

I don’t recommend using injectables*, some women can get pregnant easily after stopping it while others can’t.,, So stay on the safe side and use IUD, It is also very favorable for your age, as you are in the 30s.

*Some private clinics provide 3-month injectables usually in an unofficial way; however, the public sector remains the main source of family planning methods in Egypt.

*Male physicians, 40 years old, private clinic (scenario 1):*

IUD is one of the best methods, it remains effective for up to 10 years, safe and effective method with no side effects, it does not cause hypertension or weight gain. It is like a ring that you can wear and remove whenever you want and your ovulation is working normally.

The imposed restriction of IUD use when having a history of PID, which was incorrectly observed in previous studies, was not observed as a barrier for the use of IUD in this study. The majority of physicians counseled their clients about the negligible effect of IUD on causing PID, and that instances of PID related to IUD use were rare and typically due to poor sterilization during insertion. Moreover, they correctly mentioned that a history of PID does not automatically preclude the use of IUD use. In Assiut Governorate, IUDs were recommended for individuals with a history of PID, as the best available method for a 30-year-old woman with one or two children (second scenario). Physicians mentioned that there was no problem to use IUD as long as the clients have been treated properly and they were not currently experiencing symptoms of infection.

*Male physician, 55 years old, PHC unit (scenario 2):*

As long as you don’t have a current infection, we can insert IUD, We use sterilized procedure in insertion,,, I will also recommend you a vaginal douche that would prevent the occurrence of any infection.

#### Lack of IUD insertion skills may cause the providers to recommend other methods, despite being favored

It was found that eight out of the 30 visited physicians, five physicians in PHC units (primary health care unit “public facility”) and three physicians in private clinics, despite that these physicians recommended the use of IUD, however, were not able to provide IUD insertion service to the clients because they lack the necessary skills to insert the IUD. Instead, they asked the clients either to use other contraceptive methods such as COCs or to seek IUD insertion in other healthcare facilities, such as general hospitals or private clinics where IUD insertion services are available. It was also observed that some untrained physicians recommended the mobile family planning clinics for IUD insertion.

*Male physician, 50 years old, PHC unit:*

I recommend the use of IUD but we don’t insert it here. There is a physician that comes every 15 days that can help you or you can wait for the mobile clinic,,, If you are in a hurry you can use pills that we offer here.

#### Providers had negative bias towards hormonal contraception methods

Most providers showed negative bias towards hormonal methods, as evidenced by their recommendation of combined oral contraceptives (COCs) to only a few clients (six out of 30). Moreover, in some of those cases, this recommendation was due to the client’s hesitancy or reluctance to use IUD or the providers’ belief that IUDs are not suitable for nulliparous and those with history of PID although there are no restrictions of using any contraceptive method in such conditions, according to the WHO eligibility criteria. This negative bias may be due to the physicians’ lack of accurate information regarding the potential complications associated with the hormonal methods.

Some providers attending to nulliparous and young clients discouraged the use of COCs and injectable contraception due to concerns about the probability of causing infertility or delays in regaining fertility after discontinuation of hormonal methods. They even claimed that these methods may permanently inhibit ovulation. Some physicians advised against using hormonal methods unless the client has had enough children. However, this opposes the medical evidence that shows no restriction for nulliparity to use any method; the only irreversible method is ligation; other than that, a women will regain her fertility after stopping the method.

*Female physician, 55 years old, private clinic:*

Hormonal methods shouldn’t be used as it takes time to get pregnant again, you should not use any hormonal method if you have only one child.

*Male physician, 48 years old, private clinic:*

You will have fatigue while using the pills, the injecatbles also may cause weight loss, The implants have the same hormones as the injectables, I don’t recommend its use as these hormones delay getting pregnant after its discontinuation.

*Female physician, 55 years old, private clinic:*

When you use a hormonal method, the hormones do not leave your body easily, so this could delay pregnancy,,, you should not use hormonal methods as you have one child, you can do when you have two or three.

It was also observed that even though IUDs were recommended for most clients, the hormonal IUD was not recommended by any of the physicians despite its suitability for clients with menstrual disturbances or those fearing from the prolonged menstrual bleeding resulting from IUD use (3rd scenario). Only two physicians mentioned the availability of a hormonal IUD but did not recommend its use and stated that it should be used in older-aged women despite the fact that hormonal IUD has no age restrictions according to the WHO eligibility criteria.

*Female physician, 35 years old, private clinic:*

There is a hormonal IUD, but I don’t recommend its use, it could be favored only for women older than 45 years, as they don’t like to have menstruation.

Moreover, the majority of physicians who recommended IUD use mentioned that it is highly favorable for being a nonhormonal method, which could entail indirect prohibition of using other hormonal contraceptive methods.

*Female physician, 35 years old, PHC unit:*

The copper IUD is the best method since it is not hormonal, you can get pregnant once you remove it ,, while injectables can cause infertility.

*Male physician, 37 years old, private clinic:*

As I told you, IUD is non-hormonal,, pills and injectables are hormones, they may delay return of fertility.

### Client-related bias

#### Nulliparous clients were discouraged to use any contraceptive methods

Surprisingly, four out of eleven physicians who were visited by a nulliparous client did not recommend the use of any modern contraception method for a nulliparous client. While most physicians advised nulliparous clients to get pregnant and have a family, only three physicians advised their nulliparous clients to use alternative methods, such as the safe period, emergency contraception, or condom, as the more appropriate methods based on their age and parity status. They have clarified their concerns about the association of different contraceptive methods with infertility. Some physicians have related their objection to their ethical obligation to safeguard the health and safety of their clients. Physicians claimed that the use of IUDs increases the risk of causing intrauterine infections and intrauterine adhesions and potentially subjects the users to ectopic pregnancy, particularly in nulliparous women. Some physicians have even claimed that IUD usage in nulliparous women is contraindicated. Again, this is incorrect information given to clients and opposing the WHO eligibility medical criteria for family planning methods. However, it is important to note that medical opinions on this matter can vary. While there may be concerns regarding these potential risks, it is crucial to consult with healthcare professionals to assess individual circumstances and make informed decisions regarding the use of IUDs in nulliparous women.

*Male physician, 38 years old, private clinic:*

I may recommend IUD for clients who have children ,,,, you cannot use it; you have no kids,,,,IUD can cause inflammations and block your tubes. I only would insert it if a woman no longer wants children. If you insist on using the IUD , go to another physician ,,,I will not do it , you may never get pregnant “ talked angrily”. Any doctor that will insert it for you has no ethics or is ignorant.

*Male physician, 38 years old, PHC unit:*

You are married since four months and you did not get pregnant, so just continue as you are, without using any methods. You can have intercourse in the first 5 days after the period and in the 7 days before the next period, this would be enough for now,,if you want to delay pregnancy for a while.

Clients were advised not to use any contraceptive methods until having at least one child. Physicians perceived that nulliparous should get pregnant before using any contraceptive method to ensure their fertility and avoid the infertility-related complications of different contraceptive methods.

*Male physician, 45 years old, PHC unit:*

The biggest mistake is to use a family planning method before your first pregnancy.

*Female physician, 29 years old, PHC unit:*

Go get pregnant first, then think of family planning!!! .. We all have children and we can manage,,,, It’s a basic scientific fact that you should not use IUD or any other method now.

*Male physician, 48 years old, private clinic:*

IUD is not suitable for you as you did not give birth before, so you still have a small uterus, it may also cause intrauterine adhesions, and if this happened you may not be able to get pregnant again.

*Male physician, 48 years old, private clinic:*

Scientifically, IUD is contraindicated for a woman who did not give birth before, she may not get pregnant forever, her tubes may got blocked, IUD may prevent intrauterine pregnancy, but not the ectopic pregnancy,,, any physician who insert IUD for a nulliparous women should lose his medical license.

Only two physicians agreed to insert an IUD for nulliparous clients after the clients’ insistence to use a contraceptive method for postponing next pregnancy. Despite their hesitancy to offer a contraceptive method for a nulliparous client, they provided a non-biased correct counseling about IUD use for the nulliparous and corrected the clients’ misinformation regarding the fertility concerns in relation to IUD use.

Some physicians also believed that hormonal methods may continue to inhibit ovulation even after discontinuation. COCs were recommended by only two physicians for the nulliparous clients as most physicians believed that hormonal methods should not be used except after having enough children. That is probably why they preferred offering IUDs for nulliparous clients who insisted on using a contraceptive method.

*Male physician, 40 years old, private clinic:*

You may use contraceptive suppository half an hour before intercourse, it is the only safe method for you, it is temporary and does not have side effects,,,I may insert IUD for a woman who have at least one child, and only if she insisted to,, I would rather recommend it for a woman who have four or five children.

#### Age-related imposed restrictions for contraceptive use were associated with the parity status rather than age

It was also observed that physicians had some bias related to the age of the clients. They claimed that adolescents should be prevented from using contraception by law. Apart from this claim, they did not have any restrictions related to clients’ age. However, few physicians referred to the age above 35 as the suitable age for using IUD.

*Male physician, 35 years old, private clinic:*

IUD is difficult to insert in your age (20 years), it may also cause infections which in turn may affect your fertility, if you insist on using IUD, I would never insert it for you, just take my advice and do not use it at this age.

*Female physician, 35 years old, private clinic:*

IUD doesn’t have an age limit because it is not a hormonal method. Only hormonal methods are used after the age of 35.

However, it was clear that in some cases, rejection to recommend a contraceptive method was related to the parity status rather than age.

*Female physician, 55 years old, PHC unit:*

This age will prevent you from using any method (31y), get yourself 2 or 3 children first. You can start using contraception at the age of 35.

*Female physician, 47 years old, PHC unit:*

I can’t recommend any method for you. Not because you are young, but because you did not get pregnant yet,,,. Contraception can cause you infertility.

## Discussion

Egypt is still struggling for reducing its high fertility rate similar to other developing countries [13]. Various efforts are made to increase the contraceptive prevalence rate and eliminate barriers that can hinder clients from using family planning methods [13].

Our study revealed the existence of providers’ client and method bias towards family planning methods. Although providers seemed to have the best interest of their clients in consideration, they denied the clients’ rights of having informed choice by inducing arbitrary restrictions that have been obsolete by the updated WHO eligibility criteria for using contraceptive methods [14]. Provider bias in the provision of contraceptive methods may affect the prevalence of contraceptive use and the continuation rates and ultimately increase the unwanted births [5, 9].

Method bias was evident in our study, as providers recommended copper IUD for more than half of the mystery clients. This probably explains why the IUD constitutes the largest proportion of the method mix in Egypt [9]. The percentage of women using modern methods of family planning in 2021 reached 65%, compared to around 57% in 2014. The percentage of IUD users reaches 29% of ever-married women aged 15–49, while 20% use pills, and 10% use injectables. In comparison with the EDHS-2014, it is noted that the use of pills increased by around 4 points (16% in the EDHS-2014), while the percentage of IUD users declined by around 1 point [15]. IUD dominates not only in Egypt, but it comprises 40% or more of all modern method use in many other countries [5]. Physicians’ preference for IUD could be attributed to its proven efficacy, safety, and limited complications as compared to other methods [16, 17]. Moreover, being user independent, with limited likelihood of user error, makes IUD highly cost-effective for family planning programs in developing countries, urging physicians to recommend it and family planning program managers to support its use for women with low levels of education and method use awareness [18].

Although the outdated concerns about the association between hormonal contraception and anovulation, reducing reproductive fecundity, delayed return of fertility, or infertility, were disproved by recent studies [19, 20], it was unexpectedly observed that preference of IUD by physicians in our study was attributed to their infertility-related misconceptions about hormonal contraceptive methods. Many physicians transmitted these misconceptions to the clients during counseling and prohibited them from using hormonal methods, especially the young and nulliparous clients [20]. However, consistent with other studies [21, 22], we found that lack of providers’ skills to insert IUD may hinder recommending IUD, even when preferred by the client and recommended by physicians. A recent study conducted in Egypt has also supported this finding as it showed that 45% of physicians in primary healthcare centers reported that they faced difficulties in inserting IUD [22].

Providers were also found to impose age restrictions for the use of different contraceptive methods. In our study, providers stated that women should not use family planning methods, in general, before the age of 20, and some preferred IUD use after the age of 35. This was also reported in other studies where providers imposed minimum and maximum age restriction limits [23, 24]. The main justification for the imposed age limit was the fear of infertility complication of contraception. Previous studies have also shown that providers’ behaviors and attitudes regarding contraceptive methods were more restrictive for young clients than for older clients, for whom providers were willing to provide a wider range of contraceptives, including long-acting methods [24, 25].

Despite the evidence of eligibility of nulliparous clients to use IUD, provided by the Canadian Contraception Consensus [26] and recent studies [12, 26], most physicians in our study were resistant to offer contraception, especially IUDs, for nulliparous mystery clients. Moreover, some physicians referred to illegality of offering IUD to nulliparous women, and that it is against the ethics of medical practice. This could be attributed to their low knowledge and unfounded misconceptions about contraceptive methods and their fears of infertility as a complication of use [27, 28]. In addition, providers in other settings have shown concerns about IUD in nulliparous teens including difficult insertion, increased risk of expulsion, and discontinuation [29, 30].

Many providers have previously indicated that family planning methods are only for those who are “mothers” and are not suitable for those who have not yet had a child [31]. The consistent findings in Egypt and other developing countries point to the existence of a strong association between the physicians’ sociocultural background and their bias when offering contraception to their young clients [22–24]. In our study, many providers prohibited young clients from using contraception or offered them short-term methods while offering long-term methods only for women of proven fertility. Condoms and emergency contraceptives were recommended for nulliparous clients, despite the documented high rates of unintended pregnancies associated with their use [32]. Physicians have also advised young clients to get pregnant before using any contraception for proving their fertility, denying their right to make their own fertility decisions. We believe that physicians in Egypt, as well as in many other similar communities, are affected by the community value system of the high premium of proving fecundity. This could be tied to the gendering of women’s social roles and to the cultural primacy of maternity for ensuring a respectable adult identity, as well as social stability [33]. Therefore, the apparent concerns of physicians about the biological effects of early contraceptive use on infertility may be in part due to their entrenched community customs and norms.

### Strengths and limitations

Up to our knowledge, this is the first study to explore providers’ contraception bias in the Egyptian context. Results of the study would help to elucidate reasons of low contraception use among young clients, early methods discontinuation, and the high fertility attitudes. It may also direct the program planners not only to target clients but also to target physicians providing family planning services. However, the study does not stand without limitations. Being a qualitative study, the results cannot be generalized. Deeper investigation of the providers drivers of contraception bias in urban areas and Upper/Lower Egypt is required in further studies.

## Conclusion

In this study, providers had a clear bias towards recommending IUD rather than the other contraceptive methods, which was hindered in some cases by the lack of insertion skills. They had also bias related to the parity status and age of the clients. A clear knowledge gap was observed between what has been proven by updated research and what is brought into action, as physicians had misinformation related to the use of hormonal methods and ineligibility of IUD for the nulliparous clients.

Interventions to reduce provider bias should go beyond technical training. They should regularly receive research results and be updated with recent medical eligibility criteria of contraceptive methods use. Moreover, the sociocultural beliefs of providers that may affect their practice should be explored and addressed.

## Data Availability

Data are available from the corresponding author on reasonable request, with obscuring any data that may reveal the participants’ identity.
